# A Rare Presentation of Prostate Cancer: Psoas Muscle Metastasis Without Bone Involvement

**DOI:** 10.7759/cureus.85127

**Published:** 2025-05-31

**Authors:** Mahtab Zangui, Aye M Thida, Leen Khoury, Imad Karam, Ramtin Moradi, Aastha Baral, Maksim Agaronov, Brown Jordonna

**Affiliations:** 1 Hematology and Medical Oncology, State University of New York Downstate Health Sciences University, Brooklyn, USA; 2 Pathology, State University of New York Downstate Health Sciences University, Brooklyn, USA; 3 Pathology, State University of New York Downstate Health Sciences University/Kings County Hospital, Brooklyn, USA

**Keywords:** association: prostate cancer, incidental prostate cancer, prostate cancer, prostate cancer diagnosis, prostate cancer metastases

## Abstract

Prostate cancer is one of the most common cancers worldwide. We present a unique case of metastatic prostate cancer manifesting initially with a psoas muscle mass causing abdominal and hip pain. Computed tomography (CT) imaging revealed a large mass within the left psoas muscle with prominent pelvic and retroperitoneal lymphadenopathy, initially concerning for rhabdomyosarcoma. However, a biopsy of the psoas mass confirmed metastatic prostate adenocarcinoma. Interestingly, a nuclear whole-body bone scan and spinal MRI were negative for bone involvement or other metastases. The patient was treated with anti-androgen therapy along with abiraterone and docetaxel and had a positive serologic and radiologic response. He was also offered radiation as per the phase III Prostate Cancer Consortium in Europe-1 (PEACE-1) trial. This case underscores the importance of recognizing atypical metastatic sites, such as skeletal muscle, in prostate cancer, as they can complicate initial diagnosis and delay management. Understanding the mechanisms of rare metastatic patterns and the diagnostic approaches required is crucial for improving outcomes in prostate cancer patients.

## Introduction

Prostate cancer is the second most common cancer diagnosis in men worldwide [1]. In 2025, prostate cancer accounts for about 30% of the new cancer diagnoses in men in the United States [2]. Due to advancements in early detection and treatment, many patients achieve favorable outcomes. However, prostate cancer still significantly contributes to cancer-related deaths. According to the Global Cancer Statistics (GLOBOCAN 2022), it ranks as the eighth leading cause of cancer-related death worldwide [3].

Identifying metastasis sites is crucial not only for accurate staging and treatment but also for guiding diagnosis. The bone is known as the most common site of metastasis in prostate cancer, being involved in more than 80% of the cases. While bone involvement predominates, the proportion of patients with atypical metastases is not negligible [4].

Skeletal muscle involvement in metastatic prostate cancer is a very rare phenomenon that has only been described in a handful of case reports [5-8]. To our knowledge, it has never been reported as the initial manifestation of the disease; instead, it typically presents during disease progression and recurrence and along with bone metastasis [6,7]. Here, we discuss a rare case of metastatic prostate cancer that presented with a psoas muscle mass causing pain as the first alarming symptom. Notably, the patient’s nuclear bone scan and full-spine MRI were negative for any bone involvement, further underscoring the atypical presentation of the disease in this patient.

## Case presentation

A 57-year-old Hispanic man presented to the emergency room with a three-month history of left hip pain and left lower quadrant abdominal discomfort, which radiated to his left hip and worsened with movement and weight-bearing. His medical history was significant for primary hypertension and obesity. The physical examination at the time of presentation revealed tenderness to palpation at the left lower abdominal quadrant and a painful range of motion in the left hip. Laboratory tests were largely unremarkable, except for microscopic hematuria and elevated C-reactive protein (CRP).

**Figure 1:**
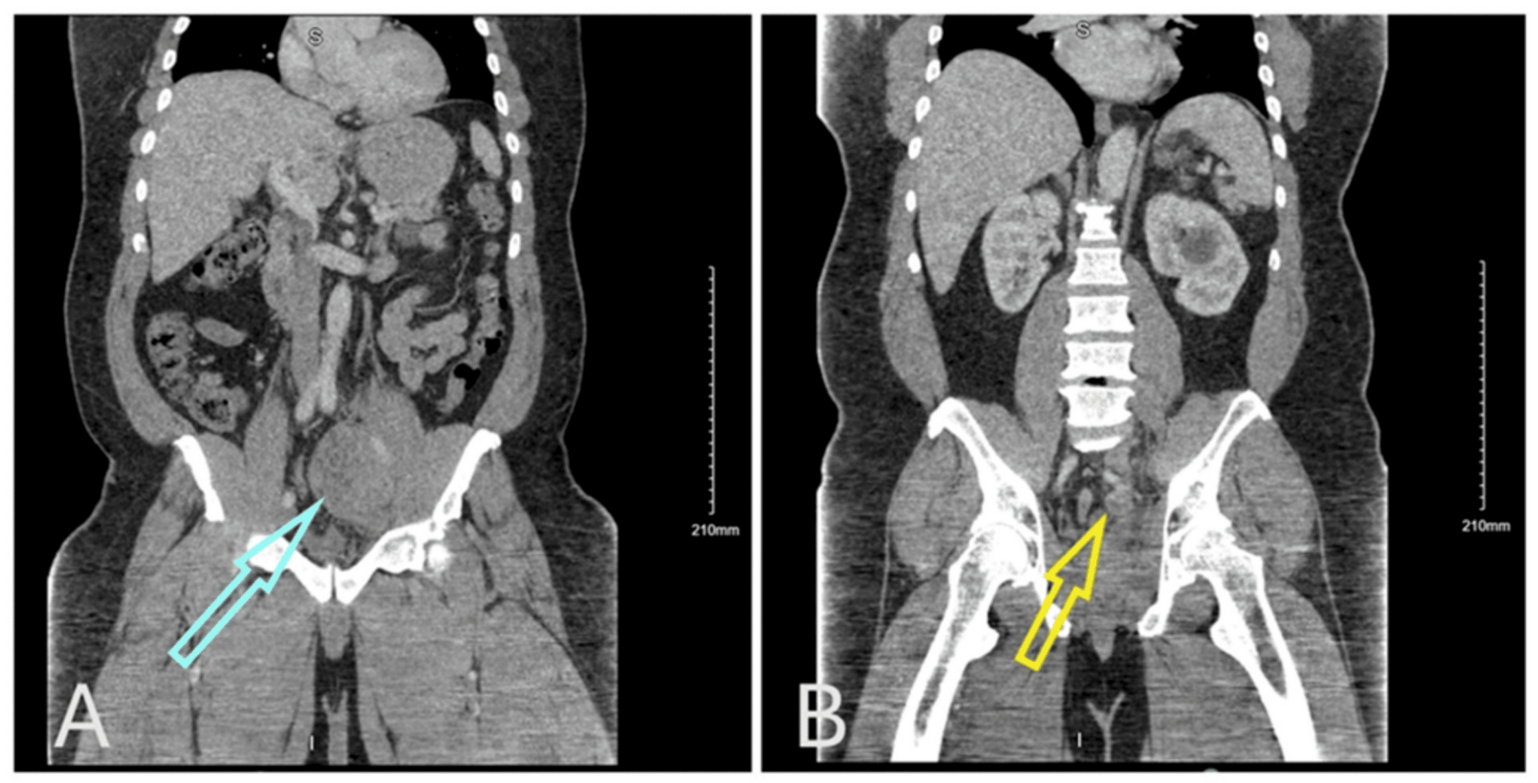
CT scan of the abdomen and pelvis. (A) A heterogeneous mass measuring 9.5 cm × 8.4 cm × 7.5 cm in the left lower quadrant (blue arrow), encasing the left ureter. (B) A smaller heterogeneous mass measuring 2.4 cm × 2.6 cm × 2.2 cm (yellow arrow) inferior to the larger mass. CT: computed tomography

X-rays of the left hip revealed moderate degenerative arthrosis but no fractures. A computed tomography (CT) scan of the abdomen and pelvis identified a heterogeneous mass measuring 9.5 cm × 8.4 cm × 7.5 cm (cranio-caudal {CC} × anteroposterior {AP} × transverse {TV}) in the left lower quadrant. This mass encased the distal left ureter and exhibited a subtle claw sign from the left psoas muscle, suggesting that it originated from the psoas muscle. Additionally, a smaller heterogeneous mass measuring 2.4 cm × 2.6 cm × 2.2 cm (CC × AP × TV) was noted inferior to the larger mass, along with prominent pelvic and retroperitoneal lymphadenopathy (Figure 1).

**Figure 2:**
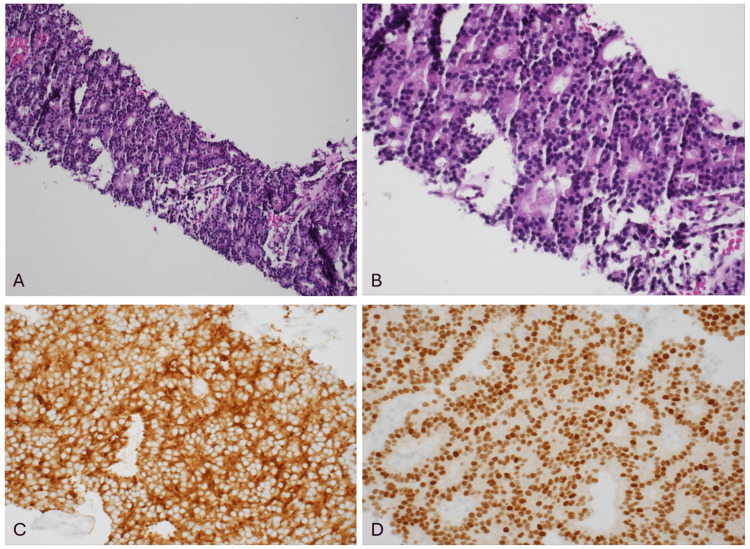
Left psoas muscle mass biopsy. (A) Hematoxylin and eosin, original magnifications, 200×. (B) Hematoxylin and eosin, original magnifications, 400×, showing gland-forming tumor cells arranged predominantly in a cribriform growth pattern. (C) The IHC PSA, original magnifications, 400×, with diffuse cytoplasmic staining highlighting the tumor cells. (D) The IHC *NKX3.1*, original magnifications, 400×, showing the strong nuclear stain highlighting the tumor cells. IHC, immunohistochemistry; PSA, prostate-specific antigen

An ultrasound-guided percutaneous biopsy of the psoas muscle mass confirmed metastatic prostate adenocarcinoma, about four weeks after the initial presentation (Figure 2). The patient’s prostate-specific antigen (PSA) level at this time was 1545 ng/mL (normal range: 0-4 ng/mL), and testosterone was measured at 396 ng/dL (normal range: 300-740 ng/dL), leading to a diagnosis of castration-sensitive metastatic prostate cancer. At the time of diagnosis, a nuclear whole-body bone scan and a full-spine MRI were negative for any bone and spine involvement. Within a few days, the patient began anti-androgen therapy with Lupron, followed by abiraterone and docetaxel. Given that the disease was confined to the pelvis, he was also offered radiation therapy in accordance with the radiation therapy arm of the phase III Prostate Cancer Consortium in Europe-1 (PEACE-1) trial.

**Figure 3:**
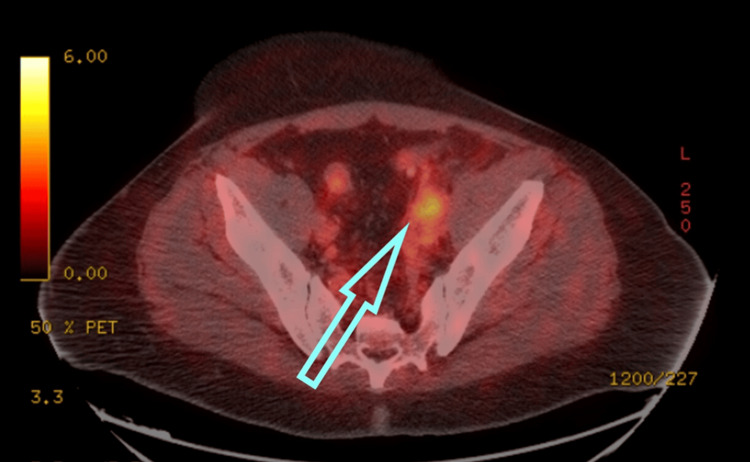
PSMA scan after the completion of the chemotherapy showing a decrease in the size of the psoas muscle mass, with residual metabolic activity (blue arrow), representing a positive response to the therapy. PSMA: prostate-specific membrane antigen

Gradually, the patient’s left hip and limping gait favoring the right side, which was apparent at initial presentation, resolved. A prostate-specific membrane antigen (PSMA) scan performed about two weeks after completing six cycles of chemotherapy indicated a decrease in the size of the psoas muscle mass, with residual metabolic activity, representing a positive radiologic response to the therapy (Figure 3). The PSA level subsequently dropped to as low as 0.08 ng/mL (normal range: 0-4 ng/mL).

## Discussion

The differential diagnosis of a psoas muscle mass includes a wide range of entities such as abscess, hematoma, primary soft tissue sarcoma, and lymphoma, making accurate diagnosis particularly challenging. Imaging findings are often nonspecific, and tissue biopsy is typically required to differentiate between infectious, inflammatory, and malignant causes. In this case, the unusual location and absence of typical metastatic sites initially did not put prostate cancer among the top differential diagnoses, which further highlights the importance of maintaining a broad differential and pursuing histopathologic confirmation when clinical suspicion remains.

Although skeletal muscle constitutes a large proportion of total body mass and receives a significant portion of cardiac output, hematogenous metastasis to skeletal muscle is exceedingly rare, with direct invasion by primary tumors being far more common. Several factors have been proposed to explain skeletal muscle’s resistance to metastatic disease, including continuous muscle motion and the mechanical disruption of tumor cells, an unfavorable pH in the microenvironment, and the muscle’s capacity to clear tumor-produced lactic acid, which otherwise promotes neovascularization in the tumor [9-12].

Metastasis to skeletal muscle has been previously reported from several primary malignancies, including pancreatic, renal, colorectal, pulmonary, gastric, and ovarian cancers [13]. However, it is extremely rare in metastatic prostate cancer, and to our knowledge, it has never been described at the time of diagnosis. The published reports are limited to disease recurrence after surgical treatment and iatrogenic tumor seeding [5-8].

On the other hand, it is known that the most common site for metastasis in prostate cancer is the bone. Based on an analysis of a large cohort of 74,826 metastatic prostate cancer patients, the most common metastasis site was the bone (84%), followed by distant lymph nodes (10.6%), the liver (10.2%), and the thorax (9.1%) [4]. This further highlights the rarity of our case, with psoas muscle metastasis presenting as the initial manifestation of the disease in the absence of any bone involvement.

From the therapeutic aspect, the PEACE-1 trial has significantly shaped the treatment landscape for de novo metastatic hormone-sensitive prostate cancer by demonstrating that the addition of abiraterone to standard androgen deprivation therapy (ADT) and docetaxel improves both radiographic progression-free and overall survival [14]. While skeletal muscle metastases are not specifically addressed due to their rarity, the trial supports early and aggressive systemic treatment in patients with high-burden or atypically presenting disease, such as in our case.

## Conclusions

This case highlights the relevance of including prostate cancer in the differential diagnosis of intramuscular lesions in older male patients, even when typical features, such as bone metastases or lower urinary tract symptoms, are absent. Clinicians should maintain a high index of suspicion for atypical metastatic presentations, particularly when initial workup is inconclusive. The early use of advanced imaging modalities, followed by image-guided biopsy when appropriate, is critical for establishing an accurate and timely diagnosis. Recognizing and reporting these rare patterns of spread can help inform clinical practice and improve early detection in similar future cases.
